# Une cause rare d’exophtalmie: l’hémangiome caverneux intraorbitaire (à propos d’un cas)

**DOI:** 10.11604/pamj.2017.26.131.9808

**Published:** 2017-03-09

**Authors:** Abderrazzak El Saqui, Mohamed Aggouri, Mohamed Benzagmout, Khalid Chakour, Mohamed El Faiz Chaoui

**Affiliations:** 1 Service Neurochirurgie, CHU Hassan II, Fès, Maroc

**Keywords:** Hémangiome caverneux, intraorbitaire, tumeur vasculaire, IRM, chirurgie, Cavernous hemangioma, intraorbital, vascular tumor, MRI, surgery

## Abstract

L'hémangiome caverneux est la plus fréquente des tumeurs vasculaires bénignes primitives de l'orbite chez l'adulte; l'âge moyen de découverte est de 42 ans avec une prédilection féminine. Cette tumeur est d'évolution lente et n'a aucune tendance à la régression spontanée; elle se localise électivement au niveau du cône musculaire en position rétro-oculaire mais pouvant se développer dans l'espace extraconique. Elle se traduit cliniquement par une exophtalmie d'apparition progressive, irréductible, non pulsatile, indolore sauf en cas de complication, associée dans 2/3 des cas à une baisse de l'acuité visuelle. Le diagnostic est facilement confirmé par l'imagerie qui permet de localiser parfaitement la tumeur par rapport au nerf optique et aux muscles oculomoteurs et de dicter la voie d'abord chirurgicale. L'exérèse chirurgicale doit être faite en bloc; elle est habituellement simple car la tumeur est bien limitée et parfaitement clivable. Les voies d'abord sont en fonction du volume et surtout du siège de la lésion. Le pronostic fonctionnel est bon et les cas de récidives sont rares.Un cas d’angiome caverneux orbitaire révélé par une exophtalmie survenant chez un patient âgé de 44 ans est rapporté.

## Abstract (en)

Cavernous hemangioma is the most frequent primary benign vascular tumor of the orbit in the adult; the median age of diagnosis is 42 years with a female predominance. This tumor develops slowly and has no tendency for spontaneous regression; it is electively located at the level of the retro-ocular muscular cone but it can develop in the extraconic space. Clinically it appears as progressive irreducible, non-pulsating, painless (unless there’s an unexpected complication) exophthalmia, associated with decrease in visual acuity in 2/3 of cases. The diagnosis is easily confirmed by imaging, which allows to precisely locate the tumor in relation to the optic nerve and the oculomotor muscles and to indicate the type of surgical approach. Surgical resection should be complete; it is usually simple because the tumor is limited and perfectly cleavable. Surgical approach is established on the basis of tumor volume and above all of the seat of the lesion. Functional prognosis is good and recurrences are rare. We here report the case of a 44-year old patient with orbital cavernous hemangioma revealed by exophthalmia.

## Introduction

Les angiomes ou hémangiomes caverneux de l’orbite sont des tumeurs vasculaires rares, représentant entre 4,5 et 7,4% de l’ensemble des tumeurs orbitaires primitives et secondaires. Néanmoins, ils sont parmi les tumeurs bénignes les plus fréquentes de l’orbite, présentant une nette prépondérance féminine; l’âge moyen de survenue se situe entre la 4^ème^ et la 5^ème^ décade de vie. Il s’agit d’une tumeur qui évolue lentement en respectant pendant longtemps la fonction visuelle et la motilité oculaire. Elle est bien encapsulée et de clivage chirurgical facile. Sa dénomination provient de « angios » et « kavernos » signifiant respectivement vaisseaux et Cavité. Cela décrit parfaitement la lésion, dont la nature est vasculaire et formée de multiples cavités sanguines ou «cavernes », de tailles variables, séparées les unes des autres par de fines parois. Tous ces éléments font que l’angiome caverneux de l’orbite a un bon pronostic fonctionnel et esthétique, malgré son siège habituellement rétrobulbaire, le plus souvent intraconique, dans une zone de grande complexité anatomique et d’abord chirurgical difficile. Nous rapportons un cas d’hémangiome caverneux orbitaire révélé par une exophtalmie survenant chez un jeune homme.

## Patient et observation

**Figure 1:**
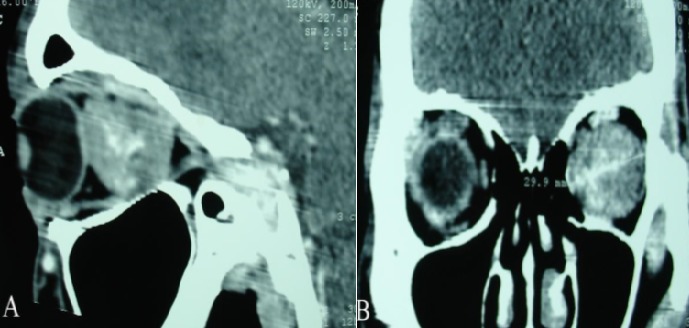
TDM orbito-encéphalique en reconstructions sagittale (A) et frontale (B) montrant un processus tissulaire intraorbitaire gauche, intraconique, refoulant le globe oculaire en avant et prenant le produit de contraste de manière hétérogène

**Figure 2:**
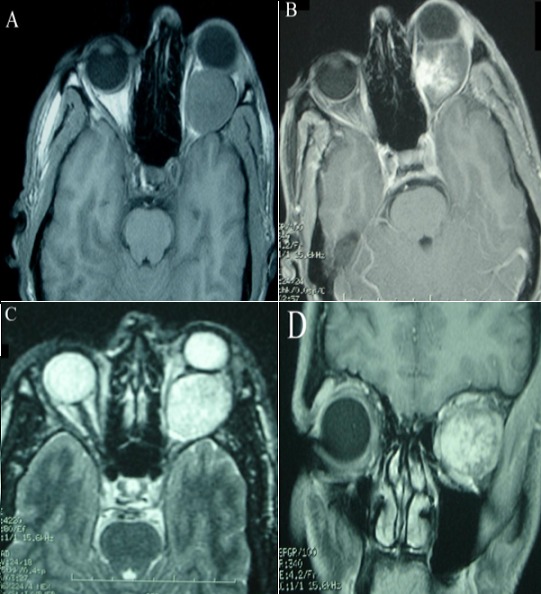
IRM orbito-encéphalique en coupes axiales T1 sans (A) et avec Gadolinium (B), coupe axiale T2 (C) et coupe coronale T1 avec Gadolinium (D) montrant un processus tissulaire intraconique gauche, bien limité, en isosignal T1, hypersignal T2, prenant fortement le contraste, refoulant le globe oculaire en avant et déterminant une exophtalmie grade III. Aspect évoquant un Angiome caverneux intraorbitaire

Il s’agit d’un homme âgé de 44 ans, sans antécédents pathologiques particuliers, qui présentait 04 ans avant son admission au service de Neurochirurgie CHU Hassan II de Fès une exophtalmie unilatérale gauche d’installation progressive avec baisse homolatérale de l’acuité visuelle, sans autres signes associés. A l’admission, l’examen clinique a trouvé un patient conscient (GCS à 15/15), sans déficit neurologique sensitivo-moteur. L’examen ophtalmologique a noté une exophtalmie axile gauche, irréductible, indolore, non pulsatile et non soufflante à l’auscultation sans signes inflammatoires en regard. L’acuité visuelle était estimée à 7/10 au niveau de l’OG et 10/10 au niveau de l’OD; la motilité oculaire était conservée et l’examen du fond d’œil a montré un œdème papillaire stade II à gauche. La TDM orbito-encéphalique a objectivé un processus tissulaire intraorbitaire gauche refoulant le globe oculaire en avant et prenant le produit de contraste de manière hétérogène (Figure 1). Le complément IRM a montré une lésion tissulaire bien limitée, en isosignal T1, hypersignal T2, prenant fortement le contraste, refoulant le globe oculaire en avant et déterminant une exophtalmie grade III (Figure 2).

**Figure 3:**
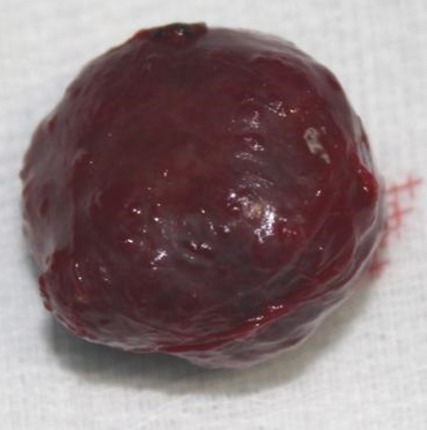
Aspect macroscopique de l’angiome caverneux enlevé en bloc

**Figure 4:**
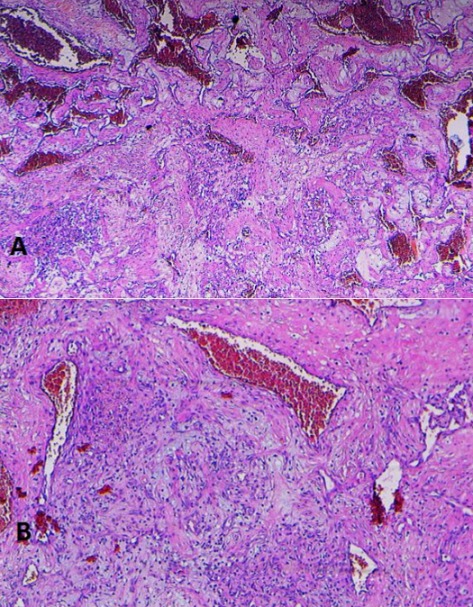
Aspect microscopique de l’ACO (A= grossissement x5, B= grossissement x140): Coupe histologique HES objectivant une prolifération vasculaire bénigne, les vaisseaux sont de taille variable, souvent dilatés congestifs et tapissés par une monocouche de cellules endothéliales régulières aplaties

Le malade a bénéficié d’une exérèse complète en bloc de la lésion intraorbitaire gauche par voie haute endocrânienne. La lésion était encapsulée, de consistance dure, refoulant le nerf optique gauche en dedans et en bas (Figure 3). L’examen histologique de la pièce d’exérèse a confirmé le diagnostic d’angiome caverneux intraorbitaire (Figure 4). Les suites opératoires immédiates étaient simples avec une régression complète de l’exophtalmie gauche et la survenue d’ecchymose palpébrale et d’un chémosis gauche ayant complètement régressé en quelques jours. Par ailleurs, le patient n’a pas présenté de ptosis postopératoire. Après un recul de deux ans, le patient a complètement récupéré son acuité visuelle à gauche et ne présentait aucun signe en faveur d’une récidive locale.

## Discussion

Les hémangiomes caverneux de l´orbite sont des tumeurs rares et représentent entre 4,5 et 7,4% de l´ensemble des tumeurs orbitaires primitives et secondaires. Néanmoins, elle demeure la plus fréquente des tumeurs primitives bénignes dans cette localisation [1]. L’âge moyen de découverte est de 42 ans, avec une prédilection dans la population féminine (70%) [2]. C’est une malformation vasculaire [3] congénitale composée de lacs sanguins localisée dans l’espace intra-conique mais pouvant se développer dans l’espace extra-conique et extra-orbitaire [4], le plus souvent profonde et inaccessible à la palpation. Cette tumeur bénigne n’a aucune tendance à la régression spontanée, contrairement aux hémangiomes capillaires de l’enfant. Macroscopiquement, elle se présente sous la forme d’une lésion ovalaire, parfois multiple, à contours réguliers, polycyclique, de couleur prune, entourée d’une capsule fibreuse. Histologiquement, l’hémangiome caverneux consiste en une large dilatation d’espaces veineux, délimité par des cellules endothéliales aplaties, encapsulées dans un tissu fibreux épais [5]. Cliniquement, les hémangiomes se manifestent par une exophtalmie [6] progressive axile ou non selon la localisation tumorale intra- ou extra-conique, non pulsatile (72% des cas), indolore, sauf en cas de complication (inflammatoire, hémorragique ou thrombotique). Le mode de découverte est parfois fortuit. Les tumeurs intra coniques compriment la face postérieure du globe et entraînent une hypermétropie. La compression du nerf optique est exceptionnelle, pouvant entraîner une baisse d’acuité visuelle [7] avec au fond d’œil des plis choroïdiens, voire un oedème papillaire.

L’imagerie contribue fortement au diagnostic. En échographie, l’hémangiome caverneux est visible sous la forme d’une masse homogène, bien limitée, hyperéchogène. Elle montre de larges espaces vasculaires contenant un flux lent bien mis en évidence par l’échographie Doppler couleur. En tomodensitométrie, la lésion est bien limitée, encapsulée, hyperdense, se rehaussant légèrement après injection, mais moins que les muscles adjacents. L’imagerie par résonance magnétique doit évaluer l’éventuel retentissement compressif, notamment sur le nerf optique. La lésion est ovalaire, bien limitée, réalisant un aspect de «globe en arrière du globe ». Elle apparaît en isosignal aux muscles en séquence pondérée T1, fortement en hypersignal en séquence pondérée T2, quasi liquidien [8], ce qui est fortement évocateur du diagnostic. La prise de contraste est également caractéristique, hétérogène, réalisant un aspect de « pommier en fleurs» [9] s’homogénéisant au temps tardif (5 minutes). Le traitement chirurgical [10] est systématique au-delà de 25 mm de diamètre, en raison de l’effet de masse sur les structures avoisinantes, notamment le nerf optique. Les récidives sont exceptionnelles et le risque de transformation maligne est nul.

## Conclusion

L’angiome caverneux de l’orbite est une tumeur bénigne rare d’évolution lente. Néanmoins, il représente la catégorie la plus fréquente des tumeurs primitives bénignes dans cette localisation. Le tableau clinique et radiologique assez stéréotypé; cependant, les moyens actuels de l’imagerie permettent une forte présomption diagnostique préopératoire surtout avec l’avènement de l’IRM.L’exérèse chirurgicale en bloc représente le traitement de choix de l’angiome caverneux de l’orbite ; elle garantit en général des résultats satisfaisants tant sur le plan fonctionnel qu’anatomique.
